# Partial Paralysis and Incontinence of Urine, Caused by Congenital Phimosis in a Boy Ten Years of Age

**Published:** 1878-11-20

**Authors:** B. M. Walker

**Affiliations:** Virginia


### PARTIAL PARALYSIS AND INCONTINENCE OF URINE, CAUSED BY CONGENITAL PHIMOSIS IN A BOY TEN YEARS OF AGE.

BY B. M. WALKER, M. D., OF VA.

In September 1877, W. M., brought his son to me for examination and treatment,stating that the boy had always wet the bed at night, and he never appeared as active as other boys, complaining of weakness of the back. I found the child weak—somewhat smaller than children of that age, with a wasted, rather senile expression. I learned from his parents that he had never had any symptoms of spasms, nor evinced signs of nervousness. Slept well, except at times this affection would cause him some anxiety (as he would say, he "was not like other children,") and not unfrequently he would have to change his night dress. This seemed *his* principal trouble from the affection.

Upon examination I found quite a long prepuce, which upon closer inspection proved to be firmly adhered from within a few lines of the meatus. He manifested no discomfort in handling it, not even when I used forible traction upon the fore-skin. He showed no reflex action from any manipulation, saying all the time that it gave him no uneasiness at all.

I told his parents that I was confident that, the patient could be relieved by an operation. They promptly gave their consent. Circumcision was performed, and I found the prepuce so adherent from the beginning, that the method usually adopted by me, to use the handle of my scalpel and break up the adhesion, would not succeed in this case, consequently I was compelled to use the point of the blade and make a very careful dissection of the entire glans; upon arriving at the corona it was possibly more adherent, or the structure better organized; as there I found it was necessary to cut *smartly*. After this came the hardened ring of smegma, which was removed, the parts were thoroughly washed in tepid water, and an oil dressing applied. Considerable inflammation supervened next day, an ointment made of oxide of zinc, Bismuth and aq-ext. opii was applied freely, covered with oil silk and upon this, cloths wrung from ice water completed the dressing; nothing unusual in the process of healing occurred. There was not as much tendency as I have observed in other cases, to re-adhere, probably this due to the precaution to smear the ointment on thickly and use retraction at least once daily.

In the due course of time the patient recovered from the effects of the operation, which proved pefectly *successful* as promised. He has not *wet* his couch ince the operation, nor is he disturbed as might he imagined to void his bladder during the night, but controls his sphincter as well as any one. As an adjuvant to overcome any tendency th.t might arise subsequently, I prescribed Ergot Iron and Tinct Canthar, to be given for a few weeks, hoping to give more tonicity the muscular structure.

I am aware this is no new treatment since the writing of Prof. Sayre, upon congenital-phimosis; still, in some respects it differs in not producing the common array of nervous phenomena so invariable in such cases. The patient rapidly gained in health, strength and activity, with a complete transformation of expression, which as intimated, was as much from *mental dijection* as anemia. His countenance is radiant with smiles, and his muscles are rapidly developing, and he is now a cheerful healthy boy. I cannot believe that any treatment, not comprising *surgical* attention, would have availed in this instance.

This may be the means of directing the younger members of the profession more particularly to a strict inspection of this organ in the cases that may occur in their practice. If so in one single instance, I shall be more than gratified for this short report.

